# Measles Outbreak Associated with a Traveler Returning from India — North Carolina, April–May 2013

**Published:** 2013-09-13

**Authors:** Kristin Sullivan, Zack S. Moore, Aaron T. Fleischauer

**Affiliations:** North Carolina Div of Public Health; Career Epidemiology Field Officer, CDC

On April 14, 2013, public health officials in North Carolina were notified of suspected measles infections in two unvaccinated members of a family. Measles was confirmed by laboratory testing at the State Laboratory of Public Health on April 16, 2013. Investigators learned that a third unvaccinated member of the household had developed fever and rash 11 days earlier, after returning to the United States from a 3-month visit to India, but measles had not been suspected until household contacts sought evaluation for similar symptoms.

During April and May, direct and indirect transmission from the returning traveler resulted in 22 identified cases of measles (including the two cases first reported), for a total of 23 cases overall. Most cases were among residents of a largely unvaccinated religious community in rural North Carolina. Eighteen (78%) of the 23 patients were unvaccinated, three (13%) had been fully vaccinated with 2 doses of measles vaccine, and two (9%) had unknown vaccination status. The 23 patients ranged in age from 1 to 59 years. Measles was confirmed by laboratory testing of specimens from 16 patients (70%). Specimens collected from eight cases were sent to the Vaccine Preventable Disease Reference Center at the Wisconsin State Laboratory of Hygiene for molecular characterization. Genotype D8, the most commonly identified measles genotype in India (1), was identified in the specimens from all eight cases.

This outbreak required extensive resources from both state and local public health agencies. Estimates provided by local health departments indicated that approximately 2,200 hours were spent on control efforts. Isolation orders were issued to 30 persons with suspected or confirmed measles infection. Investigation of the contacts of these persons led to the identification of approximately 1,000 exposed persons from various settings, including health-care facilities, schools, and community events. Contacts without evidence of measles immunity were offered postexposure prophylaxis with measles vaccine or immune globulin as indicated (2). Written quarantine orders were issued to 72 (81%) of 89 susceptible contacts who did not receive measles vaccine within 72 hours of exposure, and oral quarantine orders were issued to the remaining 17 (19%).

Although measles is no longer endemic in the United States (2), importation of measles virus continues to occur. This outbreak consumed resources from state and local public health agencies for many weeks and resulted in restrictions on the movement, through isolation or quarantine measures, of approximately 115 persons in the community. Preventing future travel-associated outbreaks in North Carolina and the United States will require maintaining high rates of immunization (particularly among travelers to areas where measles is endemic), rapid identification of cases, and swift public health response.
